# Safe Ultrasonography‐Assisted Elbow Arthroscopy Technique

**DOI:** 10.1002/atn2.70321

**Published:** 2026-10-15

**Authors:** Masato Nakai, Kazuma Miyatake, Takahiro Fujisawa, Takuma Naka, Reina Nakamura, Kagayaki Sahara, Yutaka Inaba

**Affiliations:** ^1^ Department of Orthopaedic Surgery Yokohama City University Yokohama Kanagawa Japan

## Abstract

Elbow synovectomy has traditionally been performed using direct visual observation. However, recent advancements in elbow arthroscopy have increased the popularity of arthroscopic synovectomies. This minimally invasive procedure has garnered attention because of its efficacy in halting joint deterioration while minimizing patient discomfort and invasiveness. Conversely, the technical demands of arthroscopic elbow synovectomy pose challenges, often resulting in notable complications, such as nerve damage. Considering the utility of real‐time nerve visualization for injury prevention, we suggest the integration of ultrasound guidance during surgery. Ultrasound has emerged as a pivotal tool for preventing nerve injuries, offering real‐time visualization of nerves alongside surgical instruments. Using ultrasound‐guided portals, surgeons can meticulously navigate the surgical field while safeguarding vital neural structures. Here, we describe an ultrasonography‐guided surgical method for creating the portals by confirming the surrounding anatomy.

## Graphical abstract

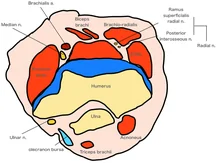

**VIDEO 1 atn270321-fig-1001:** Presentation of ultrasonography‐assisted elbow arthroscopic synovectomy. The video shows real‐time visualization of the radial and median nerves during portal creation and synovectomy, with synchronized arthroscopic and ultrasonographic monitoring to prevent iatrogenic nerve injury. Video content can be viewed at https://doi.org/10.1002/atn2.70321.

Elbow synovectomy has traditionally been performed using direct visual observation. However, recent advancements in elbow arthroscopy have increased the popularity of arthroscopic synovectomies. This minimally invasive procedure has garnered attention because of its efficacy in halting joint deterioration while minimizing patient discomfort and invasiveness.^1^, [Fig atn270321-fig-1001]

Conversely, the technical demands of arthroscopic elbow synovectomy pose challenges, often resulting in notable complications, such as nerve damage.^2^ The constrained joint cavity presents a high risk of neurovascular injuries given the proximity of crucial structures such as the brachial artery, median nerve, radial nerve, and ulnar nerve (Figure 1). Several studies have indicated a notable incidence of nerve injury during elbow arthroscopic procedures ranging from 1.7% to 4%.^3^, ^4^, ^5^ Radial nerve injury is a particularly significant concern in patients with rheumatoid arthritis because of the intricate visualization necessary for extensive synovial involvement.^6^

**FIGURE 1:**
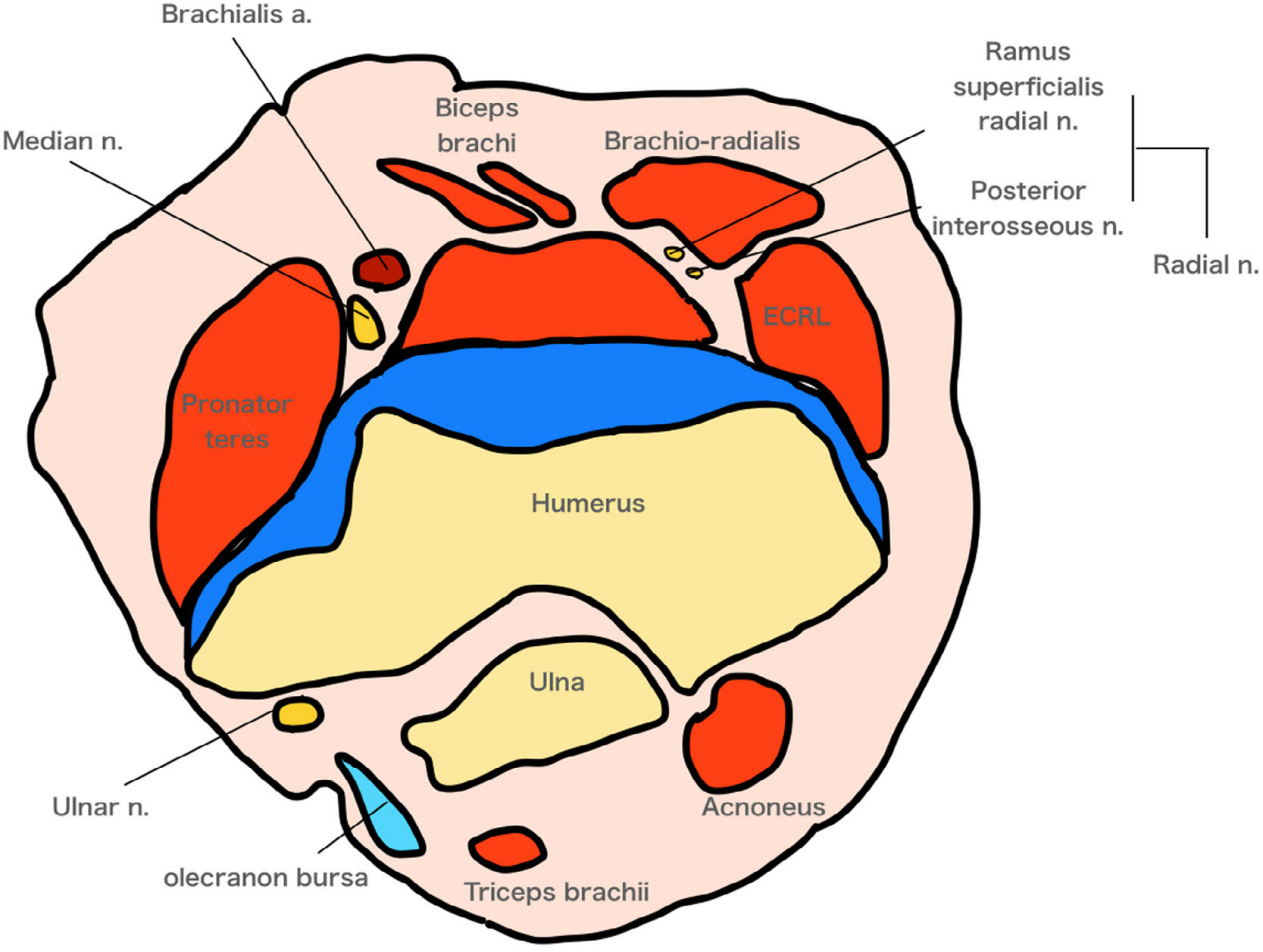
Schematic illustration showing the proximity of major neurovascular structures to the elbow joint. The BA, MN, RN, and UN are located close to the joint, indicating the potential risk of neurovascular injury during elbow arthroscopy. (BA, brachial artery; ECRL, extensor carpi radialis longus; MN, median nerve; RN, radial nerve; UN, ulnar nerve.)

Considering the utility of real‐time nerve visualization for injury prevention, we propose the integration of ultrasound guidance during surgery. Recent advancements in ultrasonography, facilitated by high‐frequency linear probes, have enabled detailed nerve visualization, prompting exploration into ultrasound‐assisted surgical techniques.^7^, ^8^

Ultrasound has emerged as a pivotal tool for preventing nerve injuries, offering real‐time visualization of nerves alongside surgical instruments, such as shavers. Using ultrasound‐guided portals, surgeons can meticulously navigate the surgical field while safeguarding vital neural structures. Herein, we describe a procedural framework that incorporates ultrasonography for precise nerve localization.

## SURGICAL TECHNIQUE

Under general anesthesia, patients are positioned supine or prone, with the elbow flexed at 90° and the shoulder abducted at 90°. Although many surgeons traditionally prefer the supine position to mitigate gravitational nerve traction, we advocate this position for the ease of transducer manipulation (Figure 2). The ultrasonography‐assisted elbow arthroscopy technique, including ultrasound‐guided portal creation and real‐time monitoring of the neurovascular structures, is shown in Video 1.

**FIGURE 2:**
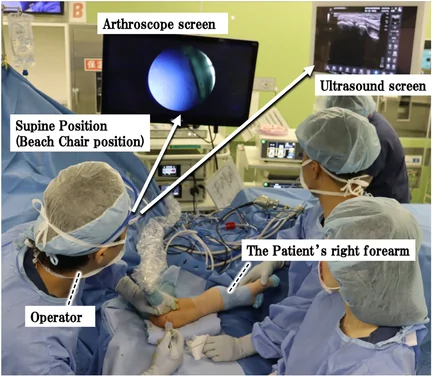
Operating room setup for ultrasonography‐assisted elbow arthroscopy. The patient is positioned in the supine beach‐chair position. The arthroscopic and ultrasound monitors are placed on the contralateral side, enabling an in‐line visualization during ultrasound‐guided needle insertion with minimal eye movement.

A bloodless operative field is achieved via tourniquet application, and a 2.7‐mm, 30° arthroscope is employed. The initial step involves establishing an anterior medial viewing portal. A 23G, 70‐mm needle serves as a guide for the skin incision, positioned 1 cm proximal to the medial humeral epicondyle and 1 cm anterior to the intermuscular septum. The integrity of the median nerve is ascertained using echography before an incision is created. After this, a 5‐mm skin incision is made along the needle's path, and the portal is enlarged to accommodate the arthroscope insertion. Subsequent confirmation of portal adequacy is attained by dynamic arthroscopic observation of radial head positioning.

Subsequently, a working portal is established. Internal delineation of the humeral and radial heads guides portal placement, with sonographic confirmation of the radial nerve location aiding needle insertion. After ensuring nerve integrity, a 5‐mm incision is made and an anterolateral portal is fashioned. Synovectomy commences under sonographic nerve guidance with shaver manipulation synchronized with arthroscopic and ultrasonographic perspectives. Notably, maintaining alignment between the camera, shaver, and ultrasonography transducer optimizes surgical precision (Figure 3).

**FIGURE 3:**
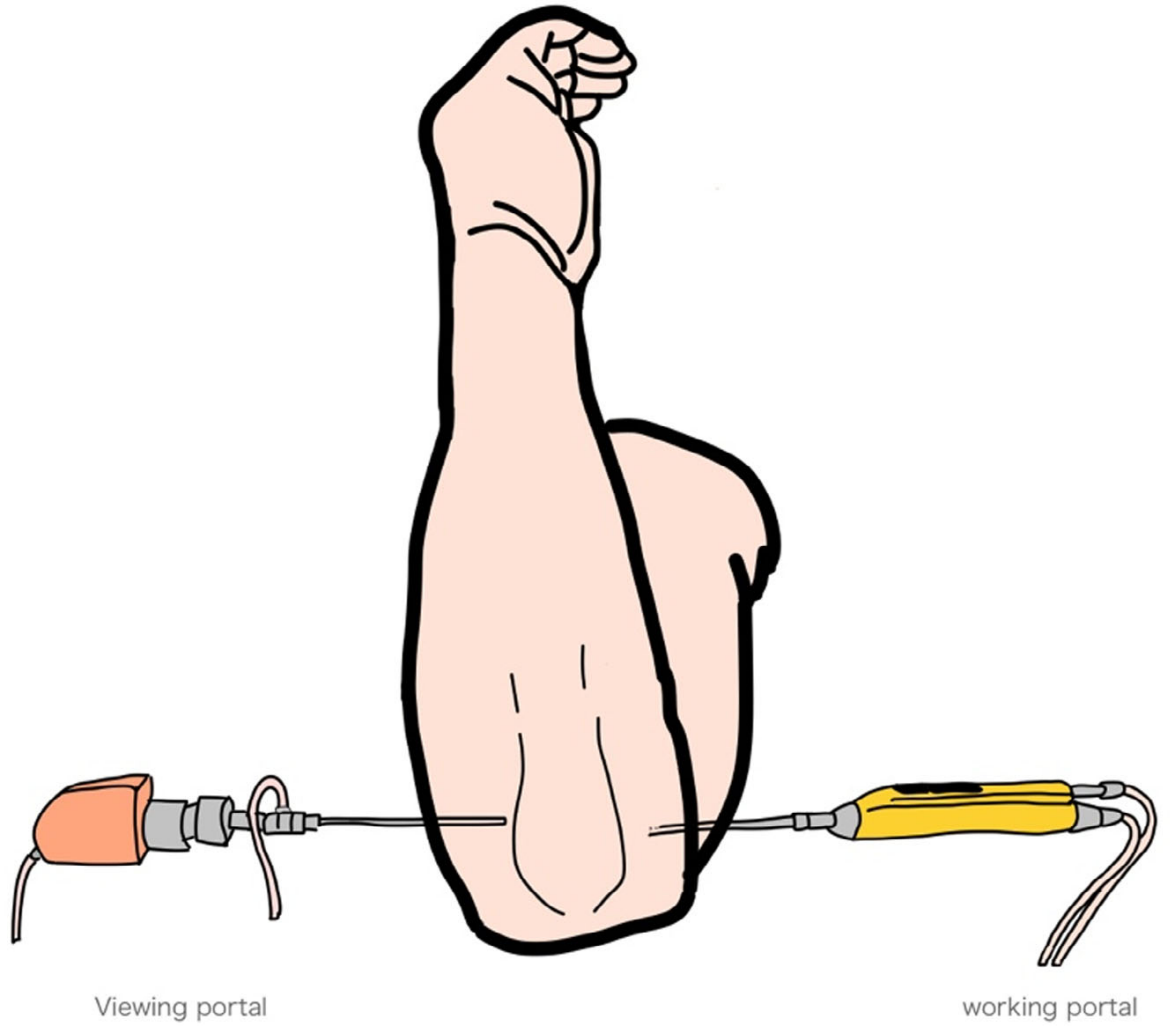
Schematic illustration of the elbow showing the arthroscopic portal configuration. The medial portal is used as the working portal, whereas the lateral portal serves as the viewing portal for elbow arthroscopy.

Synovectomy is performed after precise delineation of the nerve via sonography. This procedure is performed using a shaver. Notably, optimal alignment of the arthroscope and shaver with the echo probe enables concurrent monitoring of the instrument positions during the operation. It is imperative for surgeons to maintain awareness of this aligned configuration when establishing a portal (Figure 4).

**FIGURE 4:**
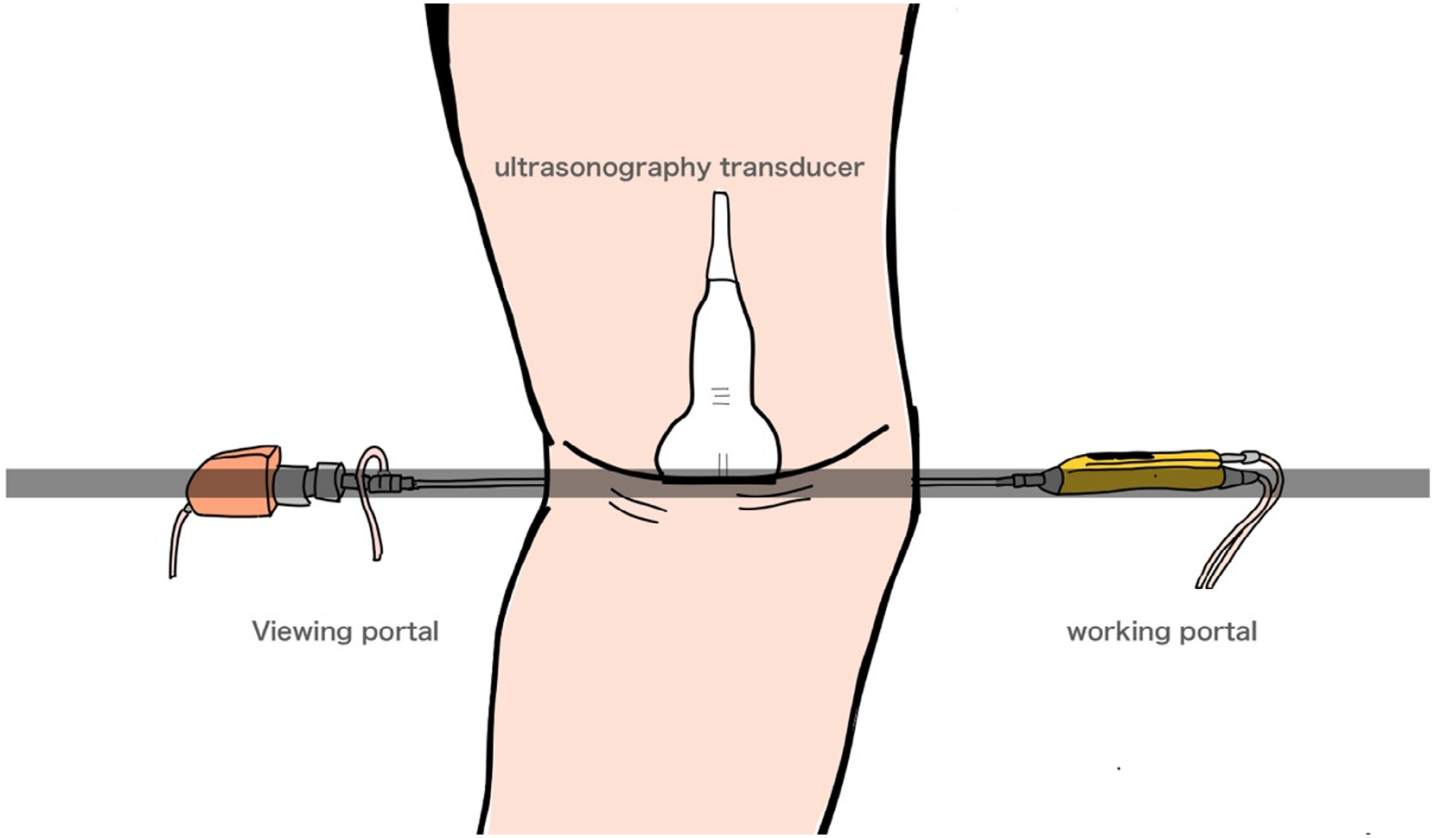
In‐line configuration for ultrasonography‐assisted elbow arthroscopy. Schematic illustration of the in‐line arrangement for ultrasonography‐assisted elbow arthroscopy. Alignment of the arthroscope, shaver, and ultrasound transducer in a single axis enables simultaneous visualization because instruments located outside the imaging plane of the transducer cannot be adequately visualized.

During synovial excision, vigilant monitoring of the nerve proximity is essential. Visualization of the radial nerve during glenohumeral synovectomy and the median nerve during ulnar synovectomy ensures nerve preservation. This integrated approach enhances the operative efficiency and minimizes the procedural duration (Figure 5).

**FIGURE 5:**
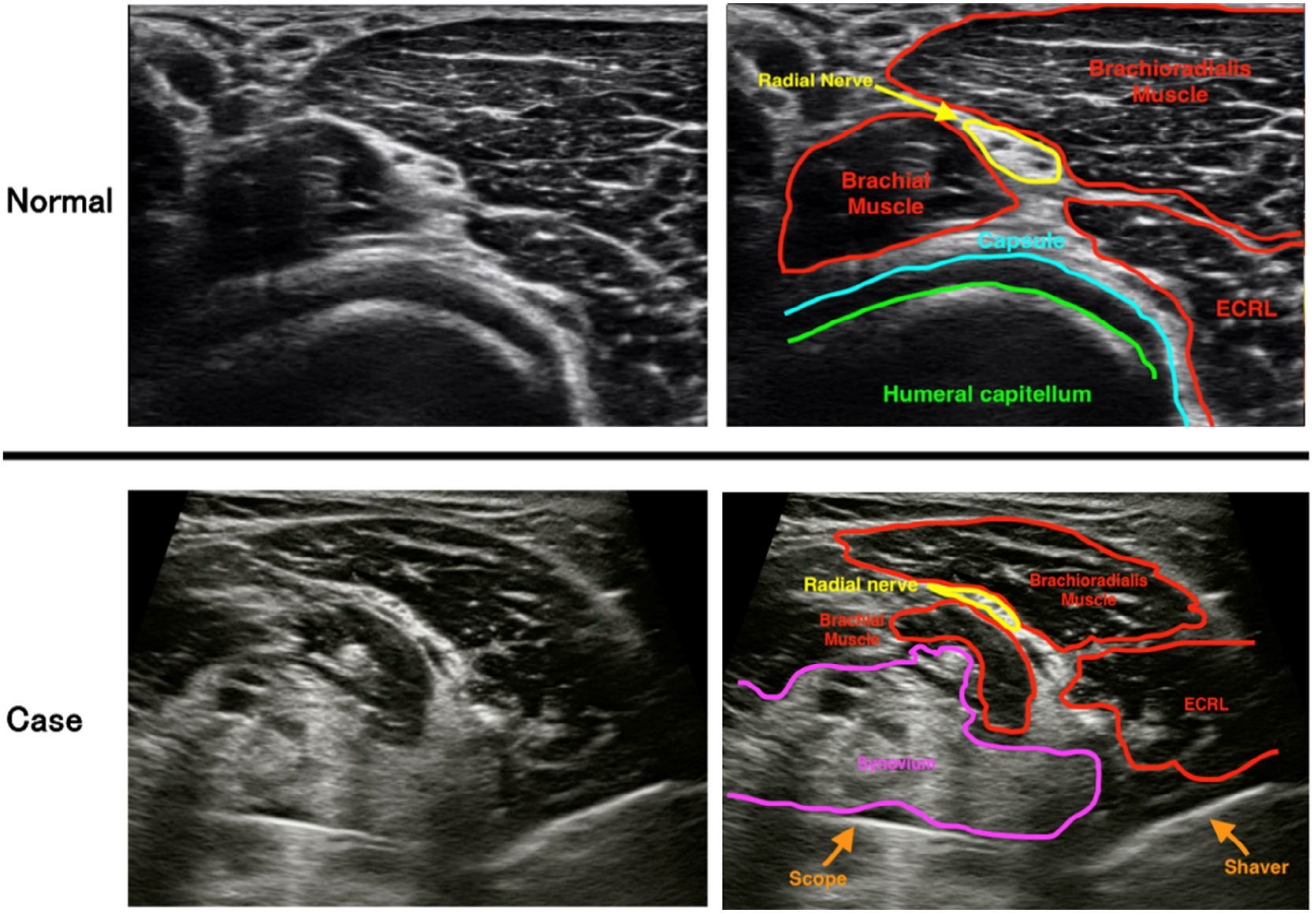
Ultrasonographic findings during elbow synovectomy. The upper row shows ultrasonographic images of a normal elbow, with clear visualization of the radial nerve. The lower row shows images obtained during synovectomy, in which the radial nerve and arthroscopic instruments are visualized within the same ultrasonographic plane, allowing real‐time monitoring of nerve proximity. This facilitates safe synovial excision and contributes to improved operative efficiency and reduced procedural duration. (ECRL, extensor carpi radialis longus.)

This facilitates the surgeon in precisely ascertaining the secure site of nerve injury during surgical intervention, thereby enhancing both the precision and expediency of the synovectomy procedure and consequently leading to a reduction in operative duration.

Key technical pearls and potential pitfalls of this technique are summarized in Table 1.

**TABLE 1:** Pearls and Pitfalls of Technique

**Pearls**	**Pitfalls**
• Precision in portal creation	• Learning curve associated with ultrasonography
• Real‐time nerve localization	• Synchronization of camera and shaver with transducer
• Visual tracking of instruments	

## DISCUSSION

### Risk of Nerve Injury With Elbow Arthroscopy

Desai et al. had conducted a survey among members of the American Society for Surgery of the Hand to elucidate complications arising from elbow arthroscopy within the preceding 5 years.^9^ Of the 372 respondents, 222 reported nerve injuries with incidences of 38%, 29%, and 22% in the ulnar, radial, and posterior interosseous nerves, respectively. Similarly, Pederzini et al. documented instances of posterior interosseous nerve palsy resulting from shaver entrapment in of 2/62 elbows, along with ulnar nerve palsy occurrences in of 2/62 elbows during specular arthrodesis for elbow contracture, necessitating subsequent surgical interventions. These patients required additional surgery.^10^

The armamentarium employed in elbow arthroscopic interventions encompasses a shaver and radiofrequency system for soft‐tissue manipulation, alongside an abrasion bar for bone resection. One potential complication is neurovascular entrapment caused by shavers.

Consequently, meticulous attention is warranted during synovectomy to avert shaver orientation toward the joint capsule instead of focusing solely on the osseous surfaces. However, reliance solely on a one‐view camera approach poses challenges in accurately discerning radial nerve depth and increases the risk of radial nerve injury. Notably, cases characterized by significant synovial hyperplasia exacerbate the risk of nerve injury owing to a compromised visual field. In selected instances, intraoperative ultrasound‐assisted radial nerve assessments revealed synovial involvement of the brachialis muscle (Figure 5), underscoring the importance of nerve injury monitoring, particularly in such scenarios.

The advantages and disadvantages of ultrasonography‐assisted elbow arthroscopy compared with conventional arthroscopic techniques are summarized in Table 2.

**TABLE 2:** Advantages and Disadvantages

**Advantages**	**Disadvantages**
• Real‐time visualization of peripheral nerves during portal creation	• Learning curve associated with intraoperative ultrasonography
• Reduced risk of iatrogenic nerve injury, particularly the radial and median nerves	• Requirement for additional equipment and personnel familiar with ultrasound handling
• Improved spatial awareness of shaver orientation relative to neurovascular structures	• Dependence on proper synchronization of arthroscope, shaver, and ultrasound transducer
• Potential reduction in operative time by preventing intraoperative complications	• Limited utility in cases with poor sonographic windows or excessive soft‐tissue swelling

Ultrasonography has emerged as a valuable adjunct for the prevention of complications, facilitating intraoperative nerve identification. Ensuring the safety of synovectomy procedures warrants meticulous confirmation of the median and radial nerves using ultrasound‐assisted surgery.

Ultrasound‐guided synovectomy represents a significant advancement in mitigating nerve injuries associated with elbow arthroscopy. By integrating intraoperative ultrasound, surgeons can confidently navigate the surgical field while preserving neural structures. This approach underscores the evolving landscape of surgical techniques aimed at optimizing patient outcomes.

## DECLARATION OF GENERATIVE AI AND AI‐ASSISTED TECHNOLOGIES IN THE WRITING PROCESS

During the preparation of this work, the authors used ElevenLabs to generate the English narration for the accompanying video. After using this tool, the authors reviewed and edited the narration as necessary and take full responsibility for the content of the publication.

## DISCLOSURES

The authors (M.N., K.M., T.F., T.N., R.N., K.S., Y.I.) declare that they have no known competing financial interests or personal relationships that could have appeared to influence the work reported in this article.
